# Diseases of Children

**Published:** 1906-04-28

**Authors:** 


### DISEASES OF CHILDREN.

(*Continued from page* 52.)

The importance of recognising the earliest manifestations of rheumatic disease in childhood is emphasised by Dr. A. E. Childs,[7] as at this stage only can one prevent by proper treatment the development of cardiac lesions. Pains in the limbs are often complained of, but their objective signs are not recognisable as in the case of adults. Wandering or growing pains, stiffness, joint pains, and articular pain, swelling, and redness are all to be regarded as different forms of rheumatism in the extremities. The last form does not usually occur until after the age of eight years. Again, a slight tonsillitis, or a little pain on swallowing due to an affection of the muscles of deglutition, or a certain stiffness of the neck may be the first manifestation of rheumatism. If with any of these signs a pulse-rate of about 120, and a temperature of 100° to 102° are present, it is probable that endocarditis has developed, even although no murmur may be audible. The diagnosis of endocarditis is supported by a blood examination if this shows a well-marked increase in the leucocytes. With reference to the differential diagnosis of rheumatism, Dr. C. G. Kerley[8] points out that the age of the patient is an important factor, for the occurrence of the disease under fifteen months is extremely rare. Rheumatism is also uncommon from the first to the third years of life, and the majority of cases occur between the fifth and tenth years. During the first fifteen months of life scurvy is often overlooked and rheumatism diagnosed, and a confirmation of the presence of the former is to be found in the absence of pyrexia, the involvement of the gums, purpura, and hæmaturia. In the early months of life joint involvement with pain and heat and constitutional disturbance almost invariably means acute epiphysitis or arthritis, due to the gonococcus or other infection. In syphilitic epiphysitis there may be pain alone without other signs. In older children muscular rheumatism is frequently diagnosed, as the result of incomplete examination, when flat foot, spinal disease, or bone or joint disease is actually present. Pain referred to the abdominal muscles is one of the earliest signs of Pott's disease. Pain complained of in the knee and inner aspect of the thigh is frequently mistaken for rheumatism.

The most common form of "habit spasm" is, according to Dr. G. F. Still,[9] a rapid blinking of the eye, or a more forcible closure of the eyelids, described by the patient as "screwing up the eyes." This was present in 47 out of 100 consecutive cases. Other movements are, a turning of the eyes, twitching of the nose, drawing up the angle of the mouth, or frowning. Sometimes more massive movements are present, such as jerking of the head or elevation of the shoulders, or some abnormal movement of the extremities. Frequently the diagnosis of the condition has to be made from the description of the parent, for the presence of the doctor has a distinctly inhibitory effect on the child, and the spasm may not occur while he is under observation. The affection is equally common amongst boys and girls, and the chief incidence of the disease is between the fifth and tenth years. Dr. Still lays stress on the association of "nervous instability" in these cases, as manifested by the accompanying disorders. These are headaches, disturbances of sleep, enuresis, irritability and fits of passion, nervous diarrhœa and convulsions. A family history of rheumatism and of neurosis is very often present. Local irritation is often the starting point of the attack. This irritation may take the form of dentition or carious teeth, or follicular conjunctivitis, or some error of refraction, or naso-pharyngeal catarrh. He suggests that habit spasm once induced by a local irritation may beget spasm elsewhere, so that a child who from some error of refraction has acquired the blinking habit may soon develop a twitching of the nose. In some cases it is possible that what is first of all a voluntary movement may by force of habit become involuntary, and may persist even after the original irritation has ceased. Important factors in the induction of the habit are mental strain and mental shock, and these amongst the poorer classes are often induced by the present system of education. The affection must be carefully distinguished from chorea, as the treatment is quite different. In cases of habit spasm, school life should be discontinued for a time, and the patient sent, if possible, to the country or the seaside. Scolding does not, in the writer's experience, do much good. Local sources of irritation should be relieved, but if an operation seems necessary, one must weigh well the question as to whether the fright of the operation may n[illegible] seriously aggravate the disturbance. As regar[illegible] drug treatment he has found most benefit from a course of bromide and arsenic; liquor arsenicalis, three to four minims with potassium bromide, five to ten grains, three times a day. Another drug which has seemed of benefit is ergot: from a half to one drachm of the fluid extract can be given with four or five minims of tincture of nux vomica three times daily.

In an article on tetany Dr. Campbell Howard[10] discusses the associated symptoms. Muscular cramps are characteristic. The spasms are most commonly seen in the hands and in the feet, but the face may be involved, producing the "risus sardonicus." Laryngo-spasm, according to Escherich and Loos, has no causal connection with rickets, and is but a special form of tetanic spasm of the respiratory muscles. Most writers are now agreed that in the majority of cases laryngismus stridulus is associated with tetany, but that a certain number of cases of laryngo-spasm form a special group independent of tetany. Trousseau's phenomenon in tetany is that pressure on the nerve trunks and blood-vessels of the affected limb will reproduce a typical spasm. The facial symptom of Chvostek is a blinking of the eye when the facial nerve on the same side is tapped at its exit from the stylomastoid foramen or along its extracranial course. Escherich describes the "mouth phenomenon," which consists in the occurrence, after tapping the corner of the mouth or the upper lip, of a contraction of the orbicularis oris on the opposite side, while at the same time the lips are brought forward as if for whistling. Erb's phenomenon is the increased excitability of the motor nerves and muscles to both faradic and galvanic stimulation. Sensory phenomena are also present in the form of pain, tenderness, formication or burning, and rarely anæsthesia. Vasomotor and trophic phenomena are seen in the solid œdema of the hands and feet, with redness of the overlying skin. Occasionally there is effusion into the tendon sheaths or joints. Erythematous, herpetiform, scarlatinal, morbilliform, and urticarial eruptions may occur.

As authorities are still at variance as to the pathology of congenital laryngeal stridor, a case reported by Dr. Koplik[11] with post-mortem examination is of interest. An infant of twelve months suffering from broncho-pneumonia and presenting the signs of congenital stridor, was admitted into hospital and died soon after from the pulmonary disease. The epiglottis was found to be curved backwards, and lay over the superior opening of the larynx. The lateral borders of the epiglottis were in contact, leaving a slit which varied from half a millimetre in its greatest extent from the tip of the epiglottis to a millimetre and a half at the arytenoid cartilages; where the space between the aryepiglottis folds was a little wider than above. The aryepiglottic folds were almost in contact, and were thin and membranous. There was no intra-laryngeal abnormality. The author thinks that this finding is opposed to the thymic theory of congenital stridor, for although the thymus was large in this patient, there was no evidence of pressure on the larynx. It is also opposed to the theory of nervous inco-ordination of Thomson and Turner, for a definite malformation was present sufficient to account for the stridor.

[7] Med. Rec., Aug. 5, 1905, p. 217. [8] Arch. of Ped., Jan., 1906, p. 47. [9] The Lancet, Dec. 16, 1905, p. 1754. [10] Am. Jour. Med. Sci., Feb., 1906, p. 301. [11] Arch. of Ped., Dec., 1905, p. 881.

